# Exploring Sources of Satisfaction and Dissatisfaction in Airbnb Accommodation Using Unsupervised and Supervised Topic Modeling

**DOI:** 10.3389/fpsyg.2021.659481

**Published:** 2021-04-21

**Authors:** Kai Ding, Wei Chong Choo, Keng Yap Ng, Siew Imm Ng, Pu Song

**Affiliations:** ^1^Department of Management and Marketing, Faculty of Economics and Management, Universiti Putra Malaysia, Seri Kembangan, Malaysia; ^2^Department of Software Engineering and Information System, Faculty of Computer Science and Information Technology, Universiti Putra Malaysia, Seri Kembangan, Malaysia; ^3^Department of Preschool Education, Guiyang Preschool Education College, Guiyang, China

**Keywords:** customer satisfaction, sharing economy, Airbnb, text mining, supervised topic modeling, big data, user-generated content

## Abstract

This study aims to examine key attributes affecting Airbnb users' satisfaction and dissatisfaction through the analysis of online reviews. A corpus that comprises 59,766 Airbnb reviews form 27,980 listings located in 12 different cities is analyzed by using both Latent Dirichlet Allocation (LDA) and supervised LDA (sLDA) approach. Unlike previous LDA based Airbnb studies, this study examines positive and negative Airbnb reviews separately, and results reveal the heterogeneity of satisfaction and dissatisfaction attributes in Airbnb accommodation. In particular, the emergence of the topic “guest conflicts” in this study leads to a new direction in future sharing economy accommodation research, which is to study the interactions of different guests in a highly shared environment. The results of topic distribution analysis show that in different types of Airbnb properties, Airbnb users attach different importance to the same service attributes. The topic correlation analysis reveals that home like experience and help from the host are associated with Airbnb users' revisit intention. We determine attributes that have the strongest predictive power to Airbnb users' satisfaction and dissatisfaction through the sLDA analysis, which provides valuable managerial insights into priority setting when developing strategies to increase Airbnb users' satisfaction. Methodologically, this study contributes by illustrating how to employ novel approaches to transform social media data into useful knowledge about customer satisfaction, and the findings can provide valuable managerial implications for Airbnb practitioners.

## Introduction

With the advancement of internet technology and the development of Web 2.0 application, the tourism and hospitality industry has experienced a significant change (Litvin et al., 2008). One of the significant changes is that an increasing number of customers are actively acquiring and sharing information on products and services on online social platforms (Podnar and Javernik, 2012) instead of solely relying on information provided by service providers. Information shared by customers on social media platforms is also regarded as user-generated content (UGC). On the internet, various types of UGC are generated, and customer reviews are considered to be the most influential UGC, which establishes a communication channel among different customers, as well as between service providers and customers (Casaló et al., 2015). For customers, online customer reviews are revealed to be an essential information source in decision-making (Shengli and Fan, 2019), a statistic indicates that almost 95% of travelers read online reviews before purchasing a travel product (Ady and Quadri-Felitti, 2015). For service providers, customer reviews reflect customers' needs and expectations, enabling novel approaches to increase customer satisfaction (Phillips et al., 2015).

In recent years, increasing attention has been paid to the importance of evaluating social media content to improve the quality of the customer experience and identify service recovery solutions, especially in the hospitality industry. Particularly, with the emergence of different text analytics, various studies have been conducted to understand customer satisfaction in the hospitality industry. For instance, Berezina et al. (2015) examined factors causing hotel customers' satisfaction and dissatisfaction by applying text network analysis. Kim et al. (2016) conducted a content analysis of online hotel reviews to identify the main drivers of hotel customer satisfaction and dissatisfaction. Xu and Li (2016) identified the determinants of customer satisfaction and dissatisfaction toward hotels through latent semantic analysis. Additionally, Padma and Ahn (2020) applied word frequency analysis to determine major themes of the luxury hotel's service quality related to hotel guest satisfaction and dissatisfaction in Malaysia. The previous studies have demonstrated the usefulness of extracting meaningful information regarding customer satisfaction from customer reviews in the hospitality industry. Some studies (e.g., Berezina et al., 2015; Xu and Li, 2016) found that the antecedents of customer satisfaction and dissatisfaction are not identical, suggesting that these two constructs can be examined separately to support strategy development for improving customer satisfaction.

Despite the wide application of text analytics in academic research, existing research on UGC in the accommodation sector of the sharing economy is probably still in its infancy (Sutherland and Kiatkawsin, 2020). Hence, this study aims to explore the sources of satisfaction and dissatisfaction in accommodations listed in a sharing economy platform, namely Airbnb, through a systematic analysis of UGC. The sharing economy is a newly developed business model based on consumption and sharing of goods and services among strangers (Gossen and Scholl, 2016). This business model has become the most rapidly developing business trends in history, with over 24 billion US dollars invested in the venture capital finding since 2010 (Wallenstein and Shelat, 2017). Airbnb is the leading example of the sharing economy in the accommodation sector (Liu and Mattila, 2017). Although both traditional hotels and Airbnb are mainly providing accommodation services, Airbnb users are often found to pursue different lodging experiences than customer choosing traditional hotels (Yi et al., 2019), such as living like a local and the opportunity to interact with the host and local community (Tussyadiah and Pesonen, 2016). Due to the distinctive preferences of Airbnb users, the results regarding customer satisfaction attributes identified from traditional hotels may not be compatible with the Airbnb context (Luo and Tang, 2019; Sutherland and Kiatkawsin, 2020).

Many studies have been conducted to understand the satisfaction of Airbnb users through the analysis of customer reviews, but previous studies (e.g., Möhlmann, 2015; Tussyadiah and Zach, 2017) only focused on satisfaction attributes and neglected dissatisfaction attributes, which leads to the assumption that the negative performance of those satisfaction attributes could be the source of Airbnb user dissatisfaction. According to Herzberg's two-factor theory (Herzberg, 1966), satisfaction and dissatisfaction are two independent continuums instead of two opposite extremes. In addition, the negative and positive performance of the same attribute would result in asymmetric impacts on customer satisfaction in the hospitality industry (Bi et al., 2019). Gu and Ryan (2008) also suggest that attributes result in customer dissatisfaction are not identical to those that lead to customer satisfaction. Therefore, in order to help Airbnb practitioners develop more effective strategies to improve user satisfaction, it is crucial to understand both guest satisfaction and dissatisfaction by examining UGC on social media (Kim et al., 2016). Kano et al. (1984) highlight the non-linear nature of customer satisfaction attributes, suggesting that service attributes contribute unequally to customer satisfaction. In order to rank the importance of service attributes in customer reviews, previous Airbnb studies (Ju et al., 2019; Luo and Tang, 2019; Ding et al., 2020) tend to be based on the emphasis level of those attributes. However, attributes with a higher emphasis level in customer reviews do not have the same level of influence on customer satisfaction (Xu, 2020a). Therefore, it is necessary to conduct further studies to determine the relative importance of identified attributes to customer satisfaction, which can help Airbnb practitioners to set priorities when developing strategies to increase customer satisfaction. Besides, this study also intends to extend previous studies by comparing Airbnb users' preferences when staying in four different types of Airbnb properties (e.g., an entire property, a hotel room, a shared room, and a private room), as Xu (2020b) found that Airbnb users staying in different types of Airbnb properties put emphasis on different aspects of the lodging experience. The findings can provide valuable suggestions for hosts to adopt more targeted strategies to ensure a satisfactory lodging experience for Airbnb users.

Given the research gap, our study aims to answer the following set of questions:

1. How to extract both satisfaction and dissatisfaction attributes from customer reviews in Airbnb accommodation?
2. How to determine the relative importance of attributes extracted from customer reviews to Airbnb users' satisfaction and dissatisfaction?
3. How do Airbnb users' review emphasis on satisfaction attributes and dissatisfaction attributes differ when they stay in different types of Airbnb properties?

To answer these questions, Latent Dirichlet Allocation (LDA), an unsupervised topic modeling technique, is employed to identify attributes that cause Airbnb user satisfaction and dissatisfaction. The results of LDA can provide proportional distributions of extracted topics in different documents, enabling us to examine how Airbnb user's perception of service attributes varies in different types of Airbnb properties. Lastly, supervised LDA (sLDA), a supervised topic modeling technique, is used to determine the relative importance of service attributes that are associated with user satisfaction and dissatisfaction.

This study makes the following theoretical contributions. First, from the perspective of methodology, this is the first study introducing the sLDA into customer satisfaction research in the lodging industry, providing a more direct solution to conduct topic regression analysis. Second, this study contributes to existing literature regarding Airbnb service improvement by enhancing our understanding of attributes resulting in both user satisfaction and dissatisfaction. As for Airbnb practitioners, this study provides valuable insights into required priority actions to increase the level of user satisfaction and specific service improvement suggestions for different types of Airbnb properties.

The rest of the paper is organized as follows. Section Literature review presents the relevant studies on Airbnb satisfaction and textual data analysis techniques. Section Methodology describes research methods and the process of data analysis. In section Results, the data and the results are analyzed. Section Conclusion concludes this study.

## Literature Review

### Airbnb Satisfaction Attributes

To increase customer satisfaction, one of the essential tasks is to identify attributes that are most related to customers' needs and expectations (Yang et al., 2011). Different sources of data have been used to identify attributes affecting the satisfaction of Airbnb users. One stream of studies used traditional survey methods (Möhlmann, 2015; Tussyadiah, 2016; Priporas et al., 2017; Lee and Kim, 2018; Sthapit et al., 2019) while another stream of studies adopted online reviews as the data source to examine Airbnb users' satisfaction (Tussyadiah and Zach, 2017; Ju et al., 2019). The major findings of these studies are summarized in Table 1.

**Table 1:** Summary of Airbnb satisfaction attributes.

**References**	**Methodology**	**Key attributes**	**Country**
Möhlmann (2015)	Survey with partial least squares technique	Utility, trust, cost savings, and familiarity.	Germany
Tussyadiah (2016)	Survey with an exploratory factor analysis	Enjoyment factors, monetary benefits (value), and accommodation amenities.	US
Priporas et al. (2017)	Survey with partial least squares technique	Service quality attributes from the questionnaire developed by Akbaba (2006).	Thailand
Tussyadiah and Zach (2017)	Text mining and regression analysis	location and feeling welcome	USA
Lee and Kim (2018)	Survey with structural equation modeling	Hedonic and utilitarian values.	USA
Sthapit et al. (2019)	Web-based survey with confirmatory factor analysis	Functional value and social value.	Italy
Ju et al. (2019)	Text mining and exploratory factor analysis	Facility service quality, visually appealing, room/house, comfortable bed, helpful host, and friendly host.	USA and Canada

Many other studies have also been conducted to identify key attributes from Airbnb user experience that could drive user satisfaction, and the findings of these studies can serve as useful references to compare with the results of the present study. Tussyadiah and Zach (2015) analyzed 41,560 reviews collected from Portland and Oregon, US, and identified three major attributes (“location,” “host,” and “property”) that frequently appeared in the Airbnb user reviews. Brochado et al. (2017) examined Airbnb user experience by analyzing 1,776 reviews collected from three countries (India, Portugal, and the USA), and eight themes were identified in this study, including “stay,” “host,” “place,” “location,” “apartment,” “room,” “city,” and “home.” Besides, Cheng and Jin (2019) identified influential attributes on Airbnb users' lodging experiences by analyzing 170,123 Airbnb reviews from Sydney, Australia, and three key influential attributes were identified in this study, including “location,” “amenities,” and “host.” Luo and Tang (2019) analyzed 250,439 Airbnb reviews collected from Los Angeles and identified five latent aspects associated with the lodging experience of Airbnb users, including “communication,” “experience,” “location,” “product/service,” and “value.”

Although online reviews have been used in previous studies to identify attributes that Airbnb users are concerned about, these studies failed to distinguish between the service attributes that determine Airbnb users' satisfaction and dissatisfaction. Moreover, most of the previous studies mainly focused on attributes that influence Airbnb users' satisfaction, but there is little research on dissatisfaction attributes. Customer dissatisfaction is an apparent reality in all the service industry. Previous studies that examine the impact of service attributes on customer satisfaction (Chowdhary and Prakash, 2005; Chen et al., 2014; Kim et al., 2016; Xu and Li, 2016; Park et al., 2020) indicate that it is insufficient to only identify one dimension of satisfaction. The assumption of one-dimensional concept is that an individual service attribute can generate both satisfaction and dissatisfaction (Oliver, 1980). This assumption has been questioned by early customer satisfaction studies suggesting that the absence of service attributes which generate satisfaction might not result in dissatisfaction (Kano et al., 1984; Cadotte and Turgeon, 1988). In addition, according to Herzberg's two-factor theory (Herzberg, 1966) that has been widely applied in customer satisfaction studies in the hospitality industry (Berezina et al., 2015; Kim et al., 2016; Tontini et al., 2017; Gerdt et al., 2019), certain attributes contributing to satisfaction do not create dissatisfaction and vice versa. More specifically, Herzberg (1966) categorized satisfaction into two distinctive groups, namely, motivation factors (satisfiers), and hygiene factors (dissatisfiers). The author suggests that missing of hygiene factors can lead to dissatisfaction, but their presence does not contribute to the enhancement of satisfaction as those attributes are perceived as guaranteed features. The realization of motivational factors can lead to the increase of customer satisfaction, but their existence does not necessarily generate satisfaction. Therefore, satisfaction and dissatisfaction are not a two-phase continuum, with one increasing and the other decreasing (Berezina et al., 2015).

Based on the Herzberg's two-factor theory, this study attempted to identify both satisfaction and dissatisfaction attributes through the analysis of Airbnb online reviews. Online reviews have been widely used as a valuable source of information to understand customer satisfaction in the hospitality industry (Guo et al., 2017). Theses reviews provide descriptions about customers' consumption experience and show their opinions toward provided services. Comparing to using customer ratings that are based on numeric figures to measure customer satisfaction (Felbermayr and Nanopoulos, 2016), customer reviews are more informative by presenting customer satisfaction and dissatisfaction in a more detailed manner (Xu and Li, 2016). In addition, in some ways, adopting the customer review based approach outperforms conventional methods, such as case studies, and survey approaches that have been heavily applied in customer satisfaction research. First, due to the open structure of customer reviews, these spontaneously generated UGC can provide more authentic reports of customers' experiences compared to surveys and case studies (Lucini et al., 2020). Second, the setting of survey questions is usually based on previous studies (Guo et al., 2017), which is not conducive to identifying novel subjects not considered in previous questionnaires, such as customer preferences in a newly developed business.

### Sentiment Analysis in Consumer Research

In the business research, sentiment analysis refers to the process of identifying different emotions (positive, negative, and neutral) toward a product or service in the text using computer-aided sentiment detection tools (Nasukawa and Yi, 2003), and these emotions were found to be strongly related to customer satisfaction. Kumar and Zymbler (2019) applied a machine learning-based sentiment analysis approach to assess customer satisfaction from airline reviews. Additionally, Zhu et al. (2020) examined the relationship between sentiment and guest satisfaction in Airbnb accommodation, adopted a lexicon-based method to classify words in each review as positive and negative, and represented guest satisfaction using accumulated online ratings. The results of this study revealed that Airbnb listings are more likely to receive higher rating scores when their reviews have a higher degree of positive sentiment. Geetha et al. (2017) examined the relationship between online hotel review sentiment and customer rating score that is used to represent the customer's satisfaction level in this study. This study concluded that the change of customer rating is influenced by reviewing sentiment polarity, and the strength of influence of review sentiment polarity on the variation of customer ratings is different in budget and premium hotels, with a value of 44 and 21%, respectively.

These studies demonstrated that sentiment polarity can be a good indicator of customers' satisfaction, and this concept has also been implemented in previous studies (e.g., Xu et al., 2017; Wang et al., 2018). After drawing insights from previous studies and considering that customer rating for individual comment is not available on the Airbnb platform, the present study used the sentiment polarity of each review as a proxy for the satisfaction level of Airbnb users; specifically, positive reviews indicate satisfaction while negative reviews indicate dissatisfaction.

In terms of techniques used to conduct sentiment analysis, lexicon-based methods based on a pre-defined list of lexical features have been widely applied in social media research (Choi et al., 2020). Considering that implementing lexicon-based methods are less time-consuming and convenient to implements, and many lexicons developed by researchers in the text mining field can be directly applied to the analysis, hence we decided to use a lexicon-based method to conduct sentiment analysis.

### Topic Modeling

Topic modeling is a machine learning-based text mining approach, which aims to reveal themes from text documents (Blei, 2012). Although human coding remains the gold standard for textual content analysis (Short et al., 2010), analyzing a large volume of customer online data is far beyond human processing capacity (Kumar and Zymbler, 2019). Topic modeling provides an automatic solution to analyze those unstructured big data without requiring manual coding, thus reducing time and costs, and also human bias. Comparing to the word-frequency based text mining approach applied in previous studies (Ju et al., 2019; Padma and Ahn, 2020), topic modeling can provide more meaningful results, as frequency-based approaches ignore the context and the underlying relationship between the words (Ahmad and Laroche, 2015).

LDA is one of the popular topic modeling techniques. The underlining assumption of the LDA model is that the words of each document result from a mixture of topics, and each topic is a distribution over the vocabulary. The outcome of LDA includes a certain number of topics represented by a list of words with a high probability of co-occurrence. Two statistical outputs are also provided in LDA, including the estimated probability of different topics within each document and the probability that a word is used to represent a topic. LDA has been widely used in consumer research to understand customers' perceptions of service. For instance, Sanchez-Franco et al. (2019) used LDA to examine the relationship between different aspects of hospitality services and service quality. Ibrahim and Wang (2019) employed LDA to extract the customers' major topics of concern to support the online retailing service improvement. Besides, LDA also provides a good solution to understand customer's satisfaction in the service-based industry. For instance, Guo et al. (2017) used LDA to identify the key dimensions of hotel customer satisfaction and analyzed 266,533 online customer reviews collected from TripAdvisor. The findings of this study revealed 19 important dimensions regarding customer satisfaction. Lucini et al. (2020) used LDA to explore dimensions of airline customers by analyzing 55,000 online customer reviews. Additionally, Bi et al. (2019) demonstrated the suitability of using LDA to extract customer satisfaction dimensions from online reviews. This study extracted 18 meaningful customer satisfaction dimensions from 25,314 electronic product reviews.

Except for LDA, there are also some other topic modeling techniques, such as structural topic model (STM) developed by Roberts et al. (2014), and dynamic topic model (DTM) developed by Blei and Lafferty (2006). Although all these topic modeling tools also can extract latent topics from the text, these tools have different focuses and require additional variables to be added in the application. For example, the emphasis of using STM is to examine the impact of pre-defined covariates on the changes of topic prevalence (Korfiatis et al., 2019). As for DTM, it is often applied to identify changes in customer perception toward certain products over time (Ha et al., 2017). Despite the availability of different topic modeling techniques, the selection of appropriate tools should be based on which of these models best fit the research objective1s (Saura, 2020). After evaluating different topic modeling techniques and referring to previous studies (Guo et al., 2017; Lucini et al., 2020), we found that LDA is more suitable for this study based on its simplicity in application and its confirmed effectiveness in customer satisfaction studies.

Although LDA is capable of identifying attributes related to customer's satisfaction from a large textual document, the results from the LDA model cannot clearly differentiate satisfaction and dissatisfaction attributes. This is because the allocation of words to each topic in the LDA model does not consider the sentiment polarity of each document, causing many topics to contain both positive and negative words. To overcome this limitation, the present study conducts LDA analysis on negative reviews and positive reviews separately, as positive topics indicating customer satisfaction have a higher probability to appear in positive reviews and vice versa (Berezina et al., 2015; Xu and Li, 2016; Hu et al., 2019). Besides, reviews with negative emotions are found to be more authentic (Chen, 2020). Therefore, a separate analysis of positive and negative reviews can shed light on the attributes that customers truly care about.

sLDA is another topic modeling approach implemented in the present study. sLDA is an extension of LDA, in which an additional response variable is modeled to determine the latent topics with the best predictive power (Blei and McAuliffe, 2007). Comparing with other topic modeling tools (e.g., STM and DTM), sLDA outperforms these tools by providing a more direct solution to conduct topic regression analysis (Blei and McAuliffe, 2007). To the best of our knowledge, sLDA has not been applied in hospitality research. In other contexts, Chai (2019) applied sLDA to analyze employee satisfaction dataset that contains both textual feedbacks and numeric ratings. The author demonstrated the effectiveness of using sLDA to extract employees' satisfaction factors that can predict satisfaction ratings. Blei and McAuliffe (2007) employed sLDA to predict movie ratings and compared the results with the LDA-based linear regression and lasso, revealing that sLDA achieved better predictive performance. In addition to sLDA, labeled LDA (_L_-LDA) is another joint topic model (Flaherty et al., 2005) while it fundamentally differs from the sLDA (Blei and McAuliffe, 2007). Kim and Kang (2018) employed _L_-LDA to determine the positive and negative attributes extracted from cosmetic reviews. In this study, customer ratings were incorporated as the document label, and the findings revealed words associated with different ratings.

For the present study, sLDA is applied to determine topics with predictive power to different levels of Airbnb user satisfaction that were represented by sentiment scores. Hence, the attributes that are most associated with Airbnb users' satisfaction and dissatisfaction can be identified. Another benefit of applying sLDA is that using more than one topic modeling techniques can generate a more meaningful analysis (Williams and Betak, 2019).

The present study differs from the previous Airbnb online reviews studies by dividing reviews into the positive and negative group. Hence, satisfaction factors and dissatisfaction factors can be clearly distinguished, and the following comparison analysis can provide additional insights into how Airbnb users' satisfaction can be increased. Besides, this study explored Airbnb reviews and their associated sentiment scores using a novel method to conduct further analysis. The joint analysis of online reviews and sentiment scores can provide us with the relative importance of different satisfaction and dissatisfaction attributes.

## Methodology

### Research Design Framework

**Figure 1:**
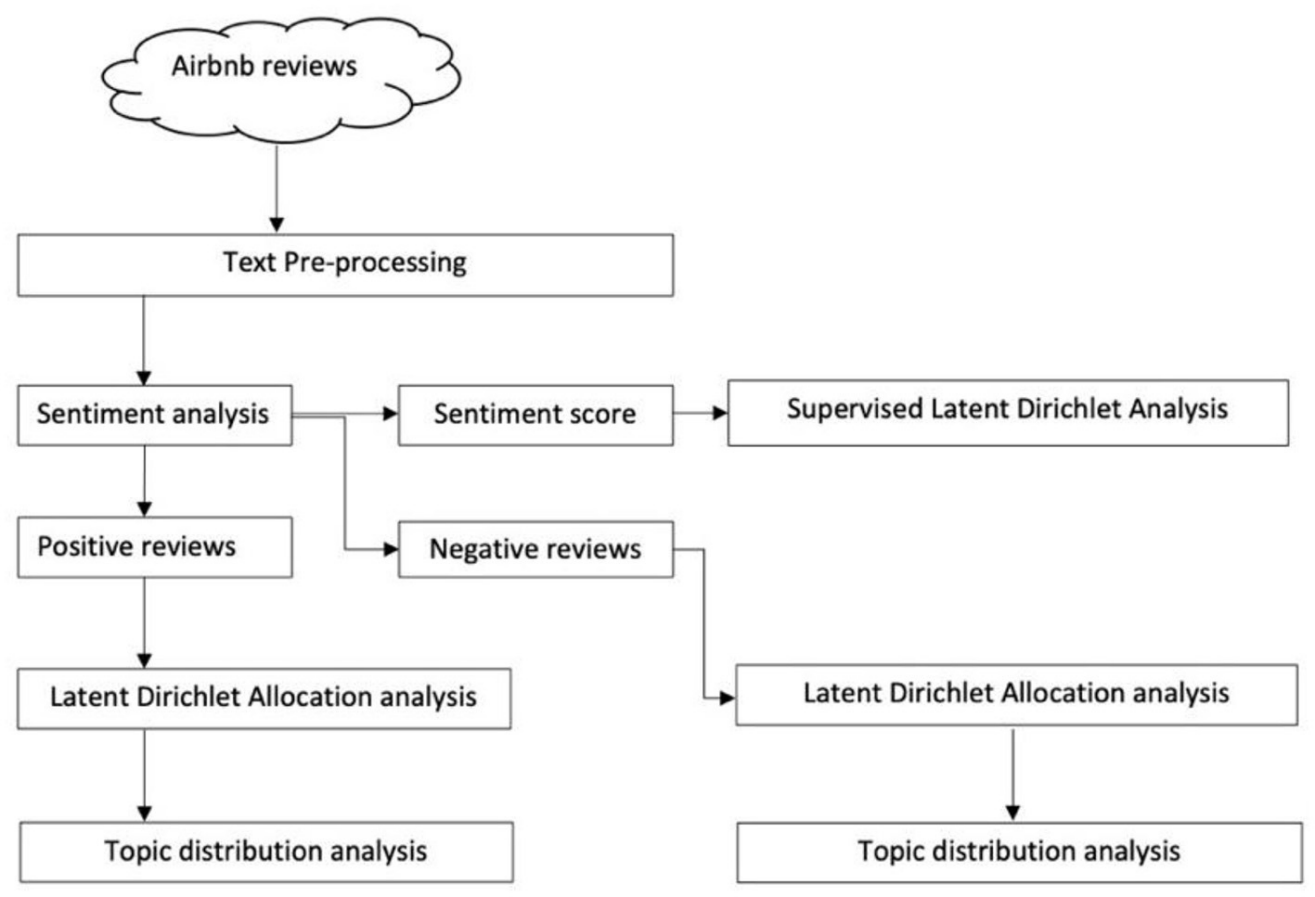
Research framework. Source: created by the authors.

The main workflow of the present study is presented in Figure 1, including the following steps: (1) collecting Airbnb review dataset; (2) pre-processing text to reduce corpus dimensions and unnecessary noise; (3) conducting sentiment analysis and re-organize the corpus; (4) extracting topics from positive and negative reviews separately using LDA; (5) examining the topic distributions in different types of Airbnb properties; (6) identifying topics that are highly associated with Airbnb user satisfaction and dissatisfaction by referring to the statistical results of sLDA.

### LDA and sLDA Method

**Figure 2:**
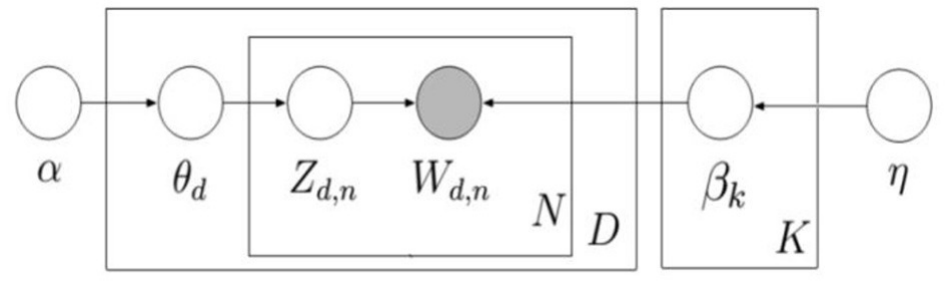
Graphical model representation of LDA (Blei et al., 2003).

Each Airbnb review is considered to be a document, and a corpus consists of *N* documents represented in *d* = (*D*_1_, *D*_2_,…, *D*_*N*_*)*. Each document is composed of *m* words represented in *D* = {*w*_1_, *w*_2_,…, *w*_*m*_}. The LDA plates are illustrated in Figure 2. *K* represents the total number of topics; β_*k*_ denotes topics distribution over different *K*; *N* refers to the number of words in a document; *D* represents the total number of the document; *Z*_*d,n*_ (per-word topic assignment) and *W*_*dn*_ (per-word topic proportions) are word-level parameters; θ_*d*_ (per-document topic proportions) denotes the document-level parameter, which is an essential metric for comparing topic distributions in different documents. Each node in the plate represents a random variable. Specifically, the node with shadow represents the observed words in the corpus, and the unshaded nodes denote the hidden variables_._ The directed edges indicate a dependence between the corresponding variables. For instance, *Z*_*d,n*_ is conditionally dependent on θ_*d*._, and *W*_*d,n*_ is conditionally dependent on *Z*_*d,n*__._ Besides, α and η are two Dirichlet parameters. In this paper, the R package “topicmodel” using Gibbs sampling is selected to perform the LDA computation.

**Figure 3:**
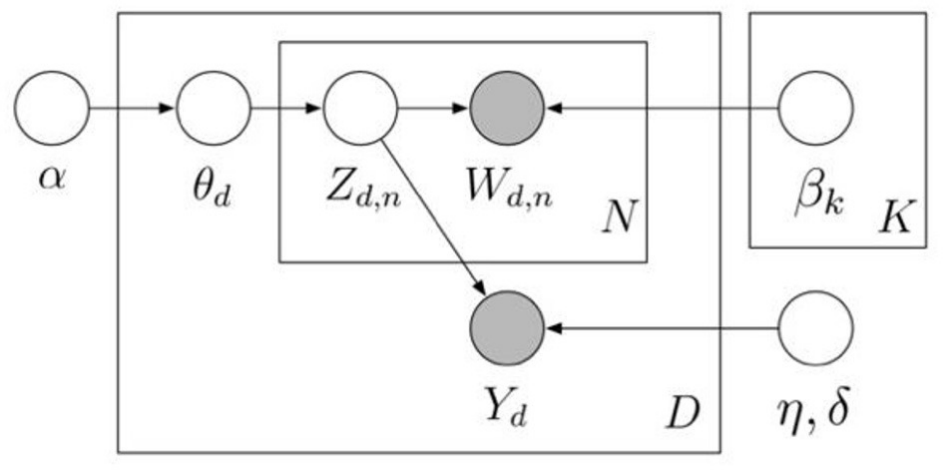
Graphical model representation of sLDA (Blei and McAuliffe, 2007).

The plate of the sLDA model is exhibited in Figure 3. In addition to adding the response variable (*Y*_*d*_) to the sLDA, there are many similarities between the sLDA and LDA. In this study, the sentiment score of each document is used as the response variable. The sentiment score (*y*_*n*_*)* of each review is modeled as:

$$\begin{array}{l} y_{n}=\lambda_{1}{\bar{z}}_{1n}+\lambda_{2}{\bar{z}}_{2\text{n}}+\ldots +\lambda_{K}{\bar{z}}_{Kn}+\;\epsilon_{n} \end{array}$$

where λ_*K*_ stands for the regression coefficients of the model; $\bar{z}$_*kn*_, *k* =1, …, *K* indicates the estimated proportion of each topic in document *n*. This regression model is fitted without an intercept, as the components of $\bar{z}$_*n*_ always sum to one (Blei and McAuliffe, 2007). Regarding notations, η and σ^2^ are pre-set constants for the normal distribution, explaining how learned topics affect the supervision (Blei and McAuliffe, 2007). Consistent with Chai (2019), the sLDA analysis was conducted in this study using the sample code in the “lda” package.

### Data Collection

The Airbnb dataset for this study was acquired from the Inside Airbnb website where the researcher can obtain publicly accessible Airbnb data for the purpose of research, and this website also provided data for several published Airbnb related studies (e.g., Horn and Merante, 2017; Cheng and Jin, 2019). We collected Airbnb online review data from 12 cities in 11 different countries from January 2012 to January 2020, with a total of 1.2 million reviews. Those cities include Beijing, Hong Kong, Tokyo, Cape Town, Paris, London, Seattle, Ottawa, Mexico City, Buenos Aires, Santiago, and Rio. The reason for selecting 12 different cities is for the purpose of improving the generalizability of the findings (Sutherland and Kiatkawsin, 2020), which is differentiated from most of previous Airbnb studies that only focus on an individual country (e.g., Cheng and Jin, 2019; Ding et al., 2020). Airbnb has expanded its business to more than 220 countries (Airbnb, 2017), and it is particularly popular for international tourists (Guttentag et al., 2018). The research results with higher generality can help Airbnb and its hosts understand some common expectations of Airbnb users and also support them to provide satisfactory services to guests from different countries. Based on the available data, we selected some well-known capitals and popular international cities from countries on different continents, where Airbnb is widely used by tourists.

### Data Pre-processing

The data pre-processing was conducted using R programming. First, all the non-English reviews were filtered using the “cld2” package. Considering that short reviews may produce an undesirable outcome because LDA is built on co-occurrence of words, reviews <50 words were excluded (Lee and Yu, 2018). The “TM” package was used to remove numbers, punctuations, and extra white space. All the words were converted to the lower case. Besides, we chose to only lemmatize all the words in the corpus (i.e., removing the inflectional ending of a word) instead of stemming words, which could result in the loss of information and poor interpretability (Hagen, 2018). Then, common stop words such as “the,” “a,” and “and” were removed, and a list of customized stop words such as city names, duplicated reviews, and system-generated phrases were also removed. Many compound words were converted as single tokens, such as “Wi Fi” to “wifi.” Moreover, words appearing <1% of the corpus were removed to reduce nosey words.

### Sentiment Analysis

After data pre-processing, sentiment analysis was performed using the R package “SentimentR” (Rinker, 2019), with zero indicating a neutral sentiment and both polarities reflecting negative and positive sentiments. Compared with other lexicon-based sentiment analysis techniques that can only classify the review as positive, neutral, and negative, one advantage of “sentimentr” is that its numeric output can be directly used to perform further regression analysis. The numeric output of sentiment analysis is used as the response variable in the sLDA model. Besides, the dictionary of “sentimentr” is incorporated with valence shifters, such as “not” and “can't,” improving the accuracy of measuring sentiment.

To ensure a balanced sample with an equal number of positive and negative reviews, we first identified the number of negative reviews in each city and then randomly selected an equal number of positive reviews in the same city. The sample summary is exhibited in Table 2.

**Table 2:** Descriptive statistics of review samples.

**Year**	**2012**	**2013**	**2014**	**2015**	**2016**	**2017**	**2018**	**2019**	**2020**	**Total**
No. of listing	297	762	1,621	3,535	5,794	8,276	11,067	13,685	2,943	27,980
No. of reviews	360	943	2,026	4,334	7,233	10,288	13,991	17,469	3,122	59,766
Entire home/apt	266	654	1,414	3,037	4,905	7,246	10,091	13,264	2,467	43,344
Private room	91	282	593	16	2,209	2,852	3,599	3,756	604	14,002
Shared room	3	3	13	1,247	78	76	100	170	15	1,705
Hotel room	0	4	6	34	41	114	201	279	36	715

**Figure 4:**
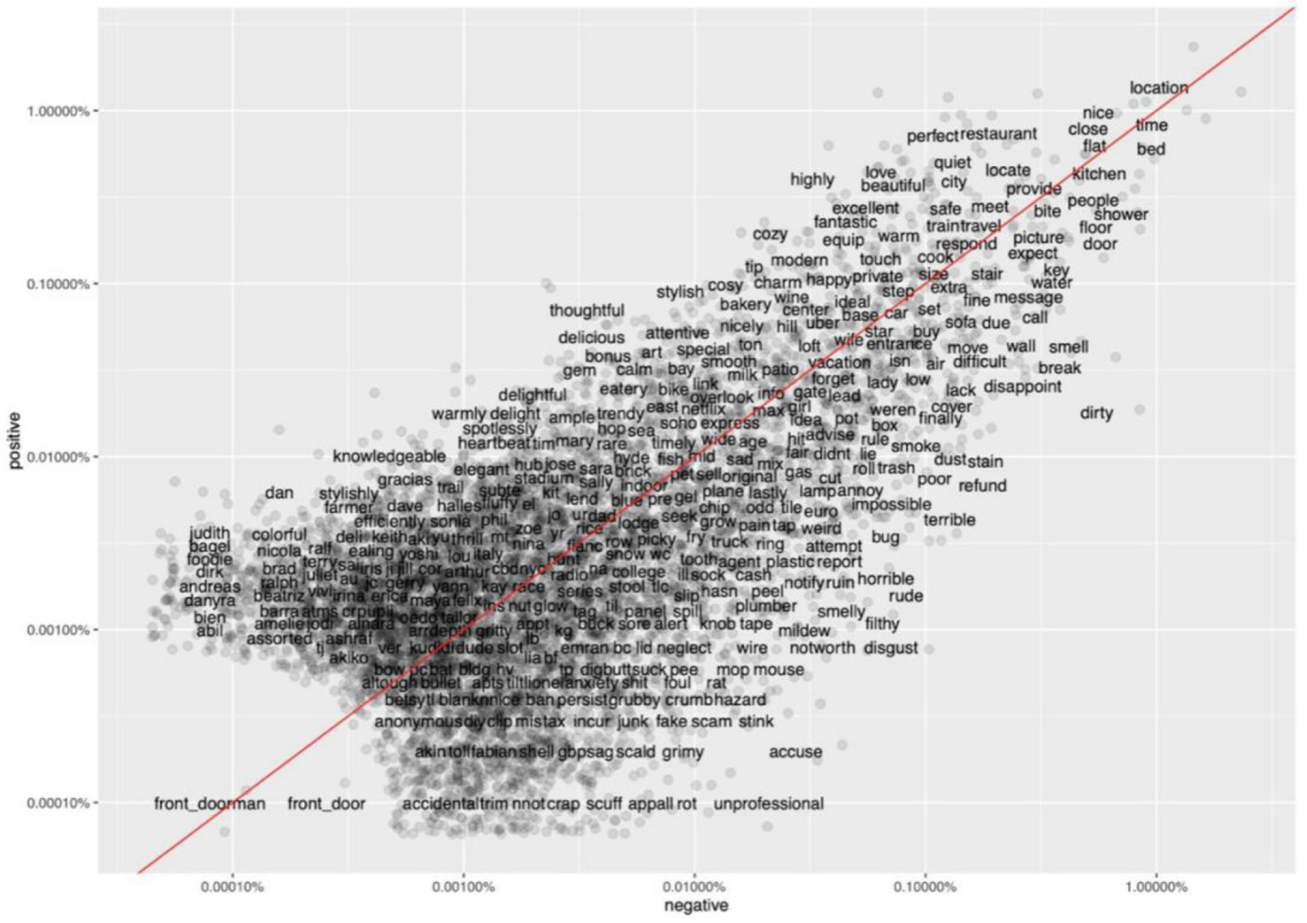
Comparison of frequent terms in positive and negative reviews. Source: created by the authors.

The comparison of frequent terms in positive and negative reviews is illustrated in Figure 4, where the horizontal axis and the vertical axis indicate the proportion of words appeared in negative and positive comments, respectively; the words close to the red line have similar frequencies in both types of reviews, such as “location,” “respond,” and “size,” and most of them are neutral words. In positive reviews, Airbnb users often compliment host behavior using words “thoughtful,” “knowledgeable,” “warmly,” and “attentive,” which rarely appeared in negative reviews. On the contrary, Airbnb hosts are often described as “rude,” “unprofessional,” and “terrible” in the negative reviews. Additionally, Airbnb users use more general words to express their feelings in positive reviews, such as “excellent,” “nice,” and “fantastic.” By contrast, negative reviews contain more words related to a specific aspect of the Airbnb lodging experience, such as “dirty,” “stain,” “smell,” “bug,” and “stink” that describe the sanitary condition of the room. Moreover, “refund” appeared significantly more and with comparatively high frequency in negative reviews. The general information regarding factors leading to Airbnb user's satisfaction and dissatisfaction is exhibited in the term frequency map, which can serve as a reference to compare with the topic modeling results.

## Results

### Topic Number Identification

In line with the research of Lee and Yu (2018) and Roque et al. (2019), the optimal topic number for LDA analysis was determined using the R package “ldatuning” **(**Murzintcev, 2015). Besides, a range of the optimal topic number can be obtained with the “ldatuning” package by running four different methods developed by Griffiths and Steyvers (2004), Cao et al. (2009), Arun et al. (2010), and Deveaud et al. (2014), respectively. As presented in Appendix A, except for the method developed by Deveaud et al. (2014), the remaining three methods indicate that more topics generate better statistical results. However, a topic model with too many topics will have a lot of overlap and duplicate keywords within the topic, making it difficult to distinguish between different topics or concepts. Therefore, the metric from Deveaud et al. (2014) metric that determines the maximum mean distance between the topic distribution pairs is followed to avoid over-clustering and reduce multiple occurrences of topics that represent the same concept; this is consistent with the study of Sutherland and Kiatkawsin (2020). After the relevance to topic results was assessed, a 14-topic solution and an 18-topic solution were selected for analyzing positive reviews and negative reviews, respectively.

### Topic Extraction and Labeling

LDA was applied to extract topics from positive reviews and negative reviews, separately, with 14 topics from positive reviews and 18 topics from negative reviews. The topic labeling is based on the group evaluation of the logic-semantic relations of top words that are most representative to each topic, as exhibited in Appendix B. Lastly, the selected topic labels were validated by analyzing the top 20 representative reviews of each topic, enabling researchers to understand the context of the top words used and then determine the appropriateness of selected topic names. The label of the topic will not be confirmed until all researchers have reached an agreement. The finalized topic names are provided in Table 3, and the identified attributes were compared with those in previous Airbnb studies. In the literature column, “Y” indicates that this attribute has appeared in the previous studies, and “N” indicates that this attribute has not been reported in the previous studies.

**Table 3:** Topic label.

**Type of reviews**	**Topic no**.	**Topic name**	**Literatures**
Positive reviews	Topic 1	Sleep disturbance	Y
	Topic 2	Help from hosts	Y
	Topic 3	Public transportation	Y
	Topic 4	Amenities	Y
	Topic 5	Location	Y
	Topic 6	Check in/out	Y
	Topic 7	View	Y
	Topic 8	Neighborhood environment	Y
	Topic 9	Room size	Y
	Topic 10	Home-like experience	Y
	Topic 11	Easy access to desired places	Y
	Topic 12	Room experiences	Y
	Topic 13	Communication	Y
	Topic 14	Revisit intention	Y
Negative reviews	Topic 1	Property issues	Y
	Topic 2	Unmatched descriptions	Y
	Topic 3	Room temperature	N
	Topic 4	Kitchen experiences	Y
	Topic 5	Noise	Y
	Topic 6	Location	Y
	Topic 7	Essay access to desired places	Y
	Topic 8	Booking and refund	Y
	Topic 9	Hosts' unpleasant behavior	Y
	Topic 10	Home-like experience	Y
	Topic 11	Door lock/key	Y
	Topic 12	Bathroom problems	Y
	Topic 13	Poor room maintenance	Y
	Topic 14	Guest conflicts	N
	Topic 15	Dirtiness and smell	Y
	Topic 16	Communication	Y
	Topic 17	Bed size/condition	Y
	Topic 18	Checking in/out	Y

### Comparative Topic Analysis

These topics were further classified into five LODGSERV dimensions (Knutson et al., 1990): tangibility, reliability, responsiveness, assurance, and empathy as presented in Table 4. The reason for choosing LODGSERV is that this instrument is specifically designed for the lodging industry to measure the customers' perception of service quality, which is regarded as the key factor to customer satisfaction. Since some topics identified in the present study are not compatible to the LODGSERV model, we followed Akbaba (2006), adding the dimension “convenience” to group those topics. In Table 4, service attributes extracted from positive and negative reviews are presented under the corresponding dimensions. In general, Airbnb users discussed significantly more on tangibility related attributes. More specifically, in the positive reviews, topics related to external environment were mentioned more often, while in the negative reviews, Airbnb users talked more about the functional failure of internal facilities. Except for the dimension of convenience, the topics under the remaining dimensions are all related to the host, which indicates the important role of hosts in Airbnb user experience.

**Table 4:** Topic classification.

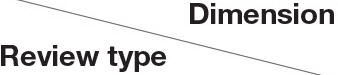	**Tangibility**	**Reliability**	**Responsiveness**	**Assurance**	**Empathy**	**Convenience**
	Amenities		Communication	Help from hosts	Home-like experience	Public transportation
	View				Check in/out	Location
Positive reviews	Neighborhood environment					Easy access to desired places
	Room size					
	Sleep disturbance					
	Room experiences					
	Property issues	Unmatched descriptions	Communication		Home-like experience	Location
	Room temperature	Booking and refund			Check in/out	Easy access to desired places
	Kitchen experiences	Hosts' unpleasant behavior				
Positive reviews	Noise					
	Door lock/key					
	Bathroom problems					
	Poor room maintenance					
	Dirtiness and smell					
	Bed size/condition					

In positive reviews, two topics are associated with the external environment: “neighborhood environment” and “views.” The other three attributes are associated with accommodation experiences: “amenities,” “room size,” and “sleeping disturbance.” In negative reviews, topics related to tangibility are mainly about different issues or problems encountered by Airbnb users. As for topic “property issues,” there are only general descriptions regarding property-related issues without mentioning a specific aspect. “Room temperature” includes Airbnb users' complaints about uncomfortable room temperature. Particularly, Airbnb users often highlighted that windows failed to regulate the room temperature. Concerning “kitchen experiences,” Airbnb users often complained about the insufficiency or poor condition of provided supplies for the cooking purpose. Besides, the topic “bathroom problems” involves Airbnb users' complaints regarding the cleanliness of the bathroom, as well as shower water temperature and pressure. “Door lock/key” includes safety concerns of Airbnb users and inconvenience in collecting the keys.

Three topics are under the dimension of reliability, and all these topics are extracted from negative reviews. The topic “unmatched description” refers to Airbnb users' dissatisfaction that listing description does not match the real situation. “Booking and refund” involves many Airbnb users' frustrations toward the booking cancellation and also some refund issues, such as delayed refund or incorrect amount of refund. As for “hosts' unpleasant behavior,” Airbnb users often complained about the irresponsible behaviors of hosts, such as failing to provide timely and effective assistance to solve the problems, inconsistent with the topic “help for hosts” from the dimension of assurance.

Under the dimension of responsiveness, the topic “communication” is extracted from both positive and negative reviews. In positive reviews, Airbnb users often highlighted the prompt reply from hosts. Besides, it can be observed from the representative reviews of this topic that the communication was mainly related to the check-in process, and Airbnb users also expressed their appreciations to clear instructions provided by the host. By contrast, “communication” extracted from negative reviews is related to Airbnb users' complaints that they are not able to reach the host or receive a timely reply from the host.

Under the dimension of empathy, “home-like experience” was extracted from both positive and negative reviews. The representative reviews of this topic reveal that Airbnb hosts provided Airbnb users home-like experiences by serving guests with great attitudes and paying close attention to their needs. In addition, Airbnb users often express gratitude to hosts for providing thoughtful daily items (e.g., soap, shampoo) and different services (e.g., breakfast, laundry). “Check-in/out” in positive reviews refers to that Airbnb hosts are able to provide flexible check-in and out services on certain occasions, such as flight delay and changing schedules. By contrast, “checking in/out” in negative reviews is often connected with Airbnb users' dissatisfaction that they are not able to check-in at a scheduled time and receive a flexible check-in or out services from the host.

Under the dimension of convenience, “location” was extracted from both positive and negative reviews, presenting Airbnb users' general descriptions about the locations; words such as “perfect,” “excellent,” “nice” were often used. Another common topic “easy access to desired places” indicates that Airbnb users care about the distance between the property and their point-of-interest, such as restaurants, shops, public transportation, and tourist attractions. Additionally, “public transportation” emerged as an individual topic in positive reviews, suggesting that satisfied Airbnb users emphasize more on transport-related convenience.

The comparative topic analysis reveals the positive and negative performance of the same attributes, demonstrating that some attributes can be the sources of both Airbnb users' satisfaction and dissatisfaction. Besides, three topics associated with Airbnb users' favorable lodging experience were extracted from negative reviews, and one topic associated with Airbnb users' complaints was extracted from positive reviews, supporting the coexistence of customer satisfaction and dissatisfaction (Chen et al., 2014).

### Topic Correlation Analysis

**Figure 5:**
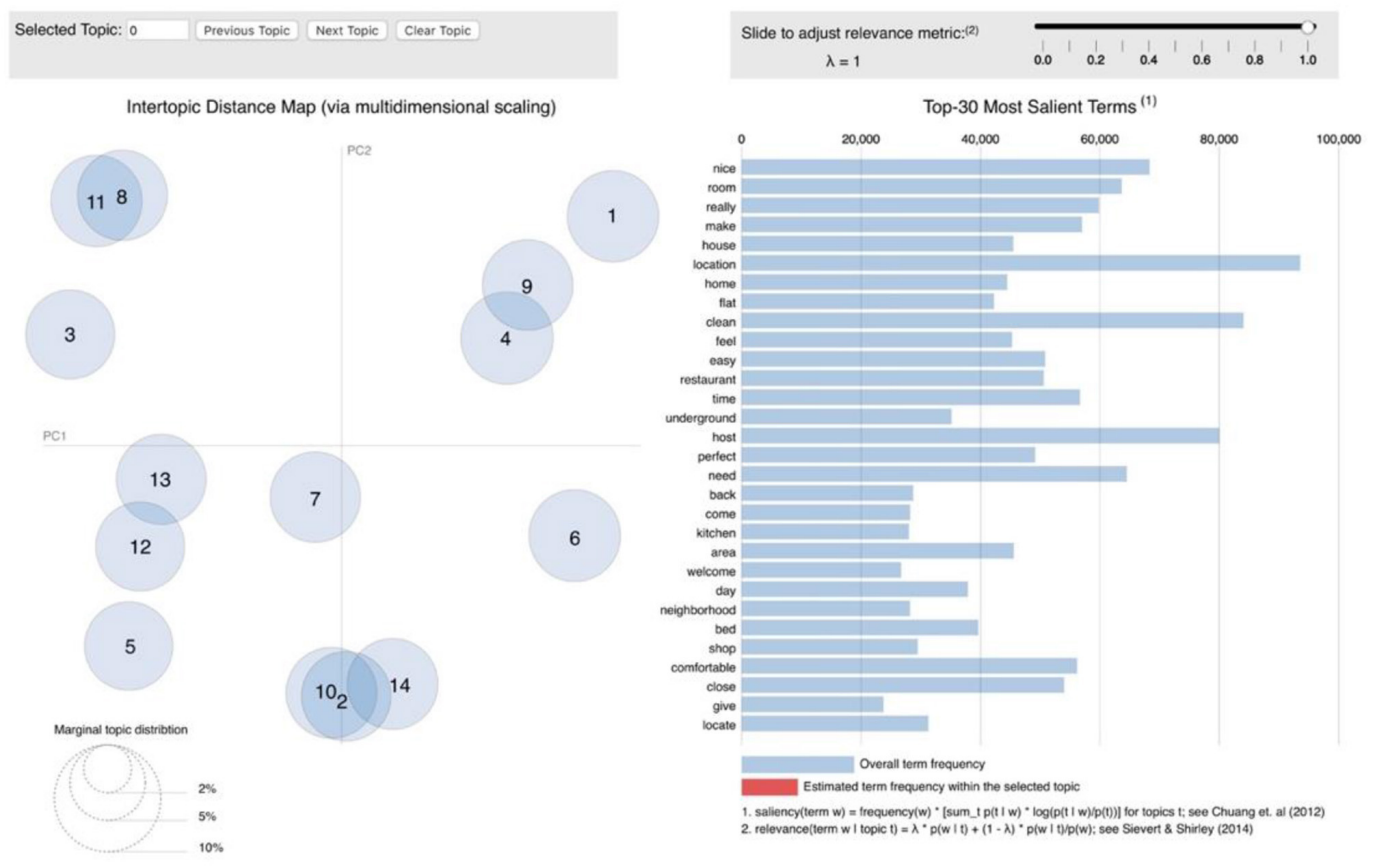
LDAvis visualization (positive reviews). Source: created by the authors.

**Figure 6:**
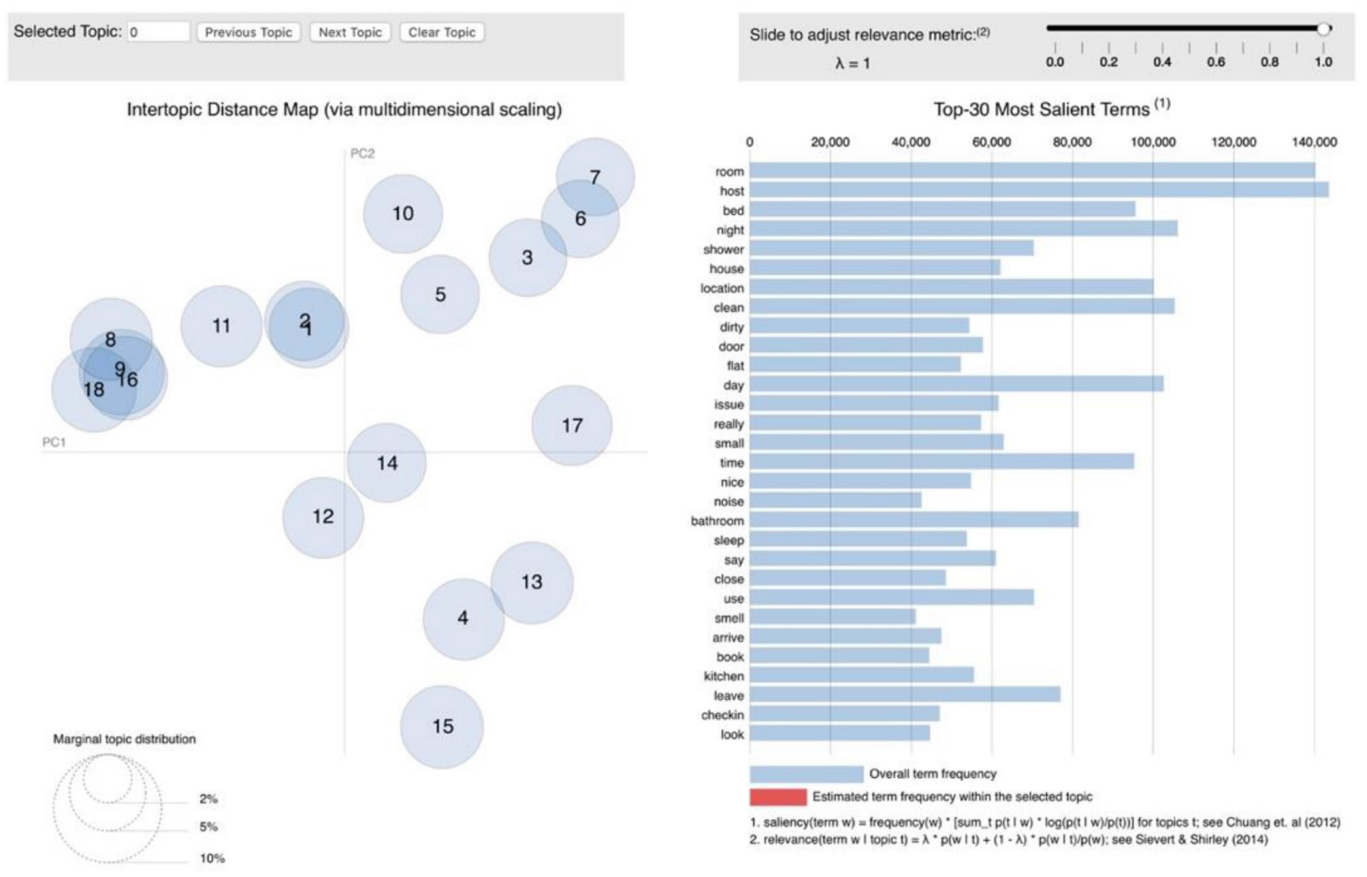
LDAvis visualization (negative reviews). Source: created by the authors.

The topic models were visualized using the R package “LDAvis” (Sievert and Shirley, 2014). “LDAvis” is a web-based interactive graphical interface in which researchers can observe the relationship between different topics generated by the LDA model and the distribution of top topic words by clicking on the corresponding topic. In this study, “LDAvis” is mainly used to reveal the relationship between different topics. In Figures 5, 6, the size of the topic circle indicates the proportional prevalence of the topic. Besides, it can be observed that the topics extracted from both positive and negative reviews shared similar proportions in the present study. The closer two topics are, the more likely they are to be discussed together in the same comment.

According to Figure 5, topic 4 (amenities) and topic 9 (room size) are closely connected because both topics contain many words related to room characteristics. Airbnb users often commented on topic 10 (home-like experience), topic 2 (help form hosts), and topic 14 (revisit intention) simultaneously. This topic cluster indicates that facilitating home-like experience and providing thoughtful help could induce Airbnb users' revisit intention. Additionally, topic 11 (easy access to desired places) and topic 8 (neighborhood environment) are closely related as both topics are related to the location of the property. Besides, the connection between topic 13 (communication) and topic 12 (room experience) is mainly because the word “host” is often mentioned in both topics.

Figure 6 indicates that topic 8 (booking and refund), topic 9 (host's unpleasant behavior), topic 16 (communication), and topic 18 (check-in/out) formed a cluster, and all these four topics contain Airbnb users' complaints about host regarding different issues, such as booking canceled by the host, bad attitude, and no response from the host during the check-in period. Besides, topic 1 (property issues) and topic 2 (unmatched descriptions) are both related to the property. The connection between Topic 3 (room temperature) and topic 6 (location) could be explained by the possibility of room temperature being uncomfortable due to geographic location. Although there is no overlap between topic 12 (bathroom problems) and topic 14 (guest conflicts), they are close to each other; consequently, guest conflicts could arise in the bathroom-related issues. The slight overlapping between topic 4 (kitchen experience) and topic 13 (poor room maintenance) indicates that poor maintenance of certain areas could result in the poor cooking experience.

### Topic Distribution Analysis

The distributions of topics extracted from both positive and negative reviews among four types of Airbnb properties were analyzed. Besides, the ANOVA test was conducted on the topic distributions, revealing that all topics extracted from both positive and negative reviews have a significant difference (*p* < 0.05) among these four types of Airbnb properties.

**Figure 7:**
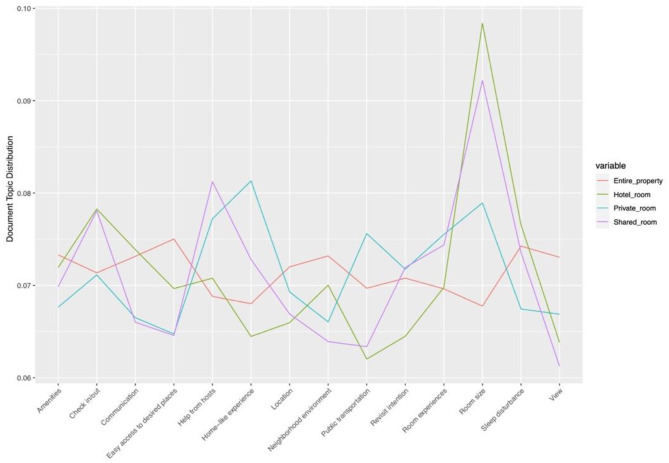
Topic distributions in different Airbnb properties (positive reviews). Source: created by the authors.

Figure 7 illustrates that Airbnb users who lived in an entire property are more likely to comment on location-related topics, such as easy access to desired places, neighborhood environment, and surrounding views; room size is much less discussed. It is noticeable that Airbnb users who chose a private room comment significantly more on the home-like experience and emphasize comparatively more public transportation. Those who lived in a shared room often write about the help from the host and room size and pay less attention to the neighborhood environment. As for the hotel room, Airbnb users comment much less on home-like experience and revisit intention but write significantly more on room size.

**Figure 8:**
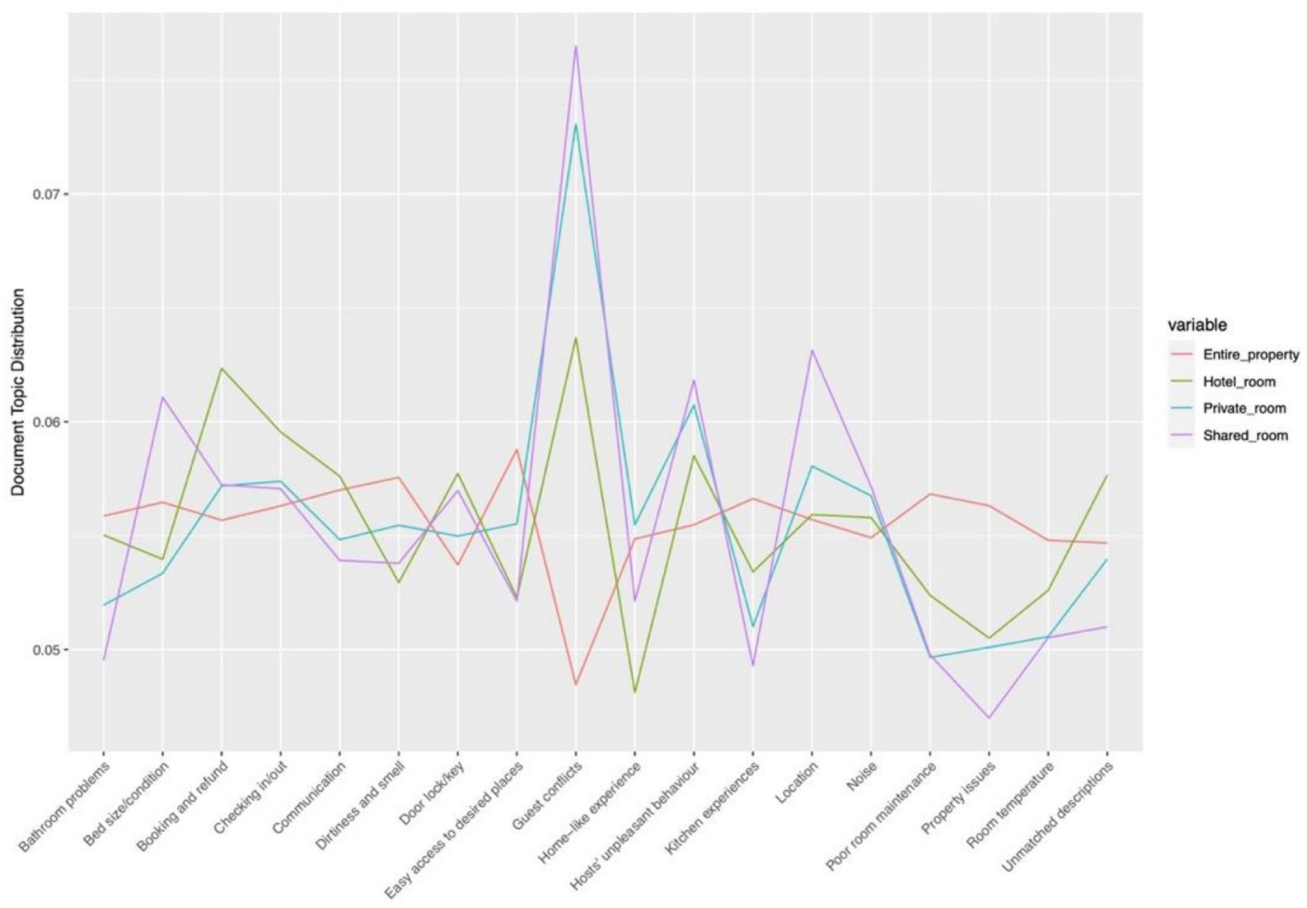
Topic distributions in different Airbnb properties (negative reviews). Source: created by the authors.

As presented in Figure 8, Airbnb users who live in an entire property complain more about bathroom problems, the sanitary condition of the room, poor kitchen experience, poor room maintenance, property issues, and uncomfortable room temperature while they are much less likely to have conflicts with other guests. Besides, those who live in a hotel room tend to complain more about issues related to booking and refund, unpleasant check-in and out experiences; they comment much less on home-like experience. However, those who chose a private room write slightly more on home-like experience. Airbnb users from the shared room and private room write significantly more on conflicts with other guests; this may be explained that Airbnb users who stay in these two types of accommodation have more opportunities to interact with other guests, and many conflicts arise from people's different hygiene habits. Additionally, those who stay in a shared room complain more about the size and condition of the bad that cause poor sleeping experience and the host's unpleasant behavior while they discuss much less on bathroom problems and property issues.

### sLDA Analysis

**Figure 9:**
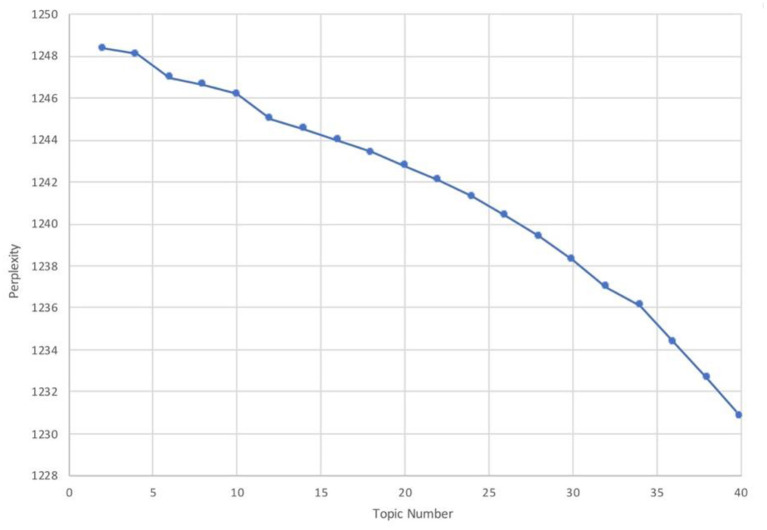
Perplexity scores of different topic solutions. Source: created by the authors.

sLDA was implemented using the “lda” package. The parameter setting is: α = 1, η = 0.1, σ^2^ = 0.25. A suitable topic number was determined by conducting test-run with different topic numbers ranging from 1 to 40. Both quantitative and qualitative criteria were used to make the final decision. The quantitative criterion refers to the perplexity of the topic model. Perplexity based on probability was used to measure how well a topic model will fit a sample (Hagen, 2018); a lower perplexity score indicates better performance. The result of the perplexity metric in Figure 9 indicates that the increase in topic number can generate a better perplexity result. However, it can be observed by examining the qualitative results that many overlapping topics in models exhibited lower perplexity score, and the logical connections of top words of some topics were not interpretable. This was because topic models with good performance in perplexity were demonstrated to be less interpretable (Chang et al., 2009). Consequently, the human judgment approach was used for the present study to evaluate the coherence and interpretability of the model. After the performance of different models was evaluated, a 20-topic solution that achieved the best semantically coherent results in the range of 1 to 40 topics was selected; it is a common topic solution when conducting topic modeling analysis in the hospitality industry (e.g., Guo et al., 2017; Hu et al., 2019). Ten top words associated with each topic are presented in Appendix C. The topic labeling process is identical to LDA. Except “guest conflicts,” the remaining topics or relevant concepts that have appeared in previous LDA analyses are also reflected in sLDA results. The topic of “guest conflicts” does not appear in the sLDA results is because that the number of words related to this topic is not significant enough to emerge as an individual topic; moreover, this topic is only strongly associated with certain types of Airbnb properties; hence, it will not affect the overall evacuation.

The sLDA analysis mainly focuses on evaluating the relationship between different topics and the review polarity score that indicates the satisfaction level of Airbnb users. Table 5 presents the estimated regression coefficient of each attribute, where a negative value indicates dissatisfaction while a positive value indicates satisfaction. Looking at Table 5, it is apparent that dirty environment of the accommodation (topic 12) is the most likely driver of dissatisfaction. At the same time, it can be seen that the host's timely reply (topic 9) and warm hospitality (topic 2) are the most important drivers of satisfaction.

**Table 5:** sLDA statistical summary.

**Topic no**.	**Coefficient**	**Estimate std. error**	***t*-value**	**Pr(>|t|)**
Topic 1	−0.318495	0.007493	−42.508	<2e-16***
Topic 2	0.767366	0.006205	123.673	<2e-16***
Topic 3	−0.244690	0.007951	−30.774	<2e-16***
Topic 4	−0.174047	0.008800	−19.778	<2e-16***
Topic 5	−0.016144	0.008573	−1.883	0.0597 .
Topic 6	0.513106	0.006926	74.086	<2e-16***
Topic 7	−0.051490	0.008101	−6.356	2.08e-10***
Topic 8	0.476621	0.008196	58.153	<2e-16***
Topic 9	0.884127	0.006776	130.470	<2e-16***
Topic 10	−0.083269	0.008994	−9.258	<2e-16***
Topic 11	−0.195362	0.008295	−23.551	<2e-16***
Topic 12	−0.456692	0.006571	−69.500	<2e-16***
Topic 13	0.074721	0.008926	8.371	<2e-16 ***
Topic 14	0.324420	0.007145	45.405	<2e-16***
Topic 15	−0.251838	0.008823	−28.544	<2e-16***
Topic 16	0.061481	0.008435	7.289	3.17e-13***
Topic 17	0.459367	0.006850	67.059	<2e-16***
Topic 18	0.406608	0.006090	66.769	<2e-16***
Topic 19	−0.033446	0.007920	−4.223	2.42e-05***
Topic 20	−0.296729	0.006919	−42.887	<2e-16***

Signif. codes: 0 “***” 0.001 “**” 0.01 “*” 0.05 “.” 0.1 “ ” 1. Residual standard error: 0.1432 on 59,746 degrees of freedom, Multiple R-squared: 0.735. Adjusted R-squared: 0.7349. F-statistic: 8,285 on 20 and 59,746 DF, p-value: <2.2e-16.

It can be observed that nine out of the 20 topics have a positive value of the coefficient and the other 11 topics have a negative value of the coefficient. As for statistical significance, except for topic 5, the remaining topics are significantly related to review polarity score. Topic 5 is at the 5.97% level, suggesting that topic 5 is not statistically relevant to Airbnb users' satisfaction. Topic 9 has the highest coefficient value of 0.884, followed by topic 2 with a coefficient value of 0.767, demonstrating that the improvement of these two aspects is most likely to increase Airbnb users' satisfaction. Regarding topics with a negative value of the coefficient, topic 12 has the lowest coefficient value of −0.4567; thus, the improvement of the sanitary condition of the room should be prioritized to prevent the Airbnb users from being dissatisfied. Another two leading causes of dissatisfaction are topic 1 and 20, with coefficient values of −0.318 and −0.297 respectively.

**Figure 10:**
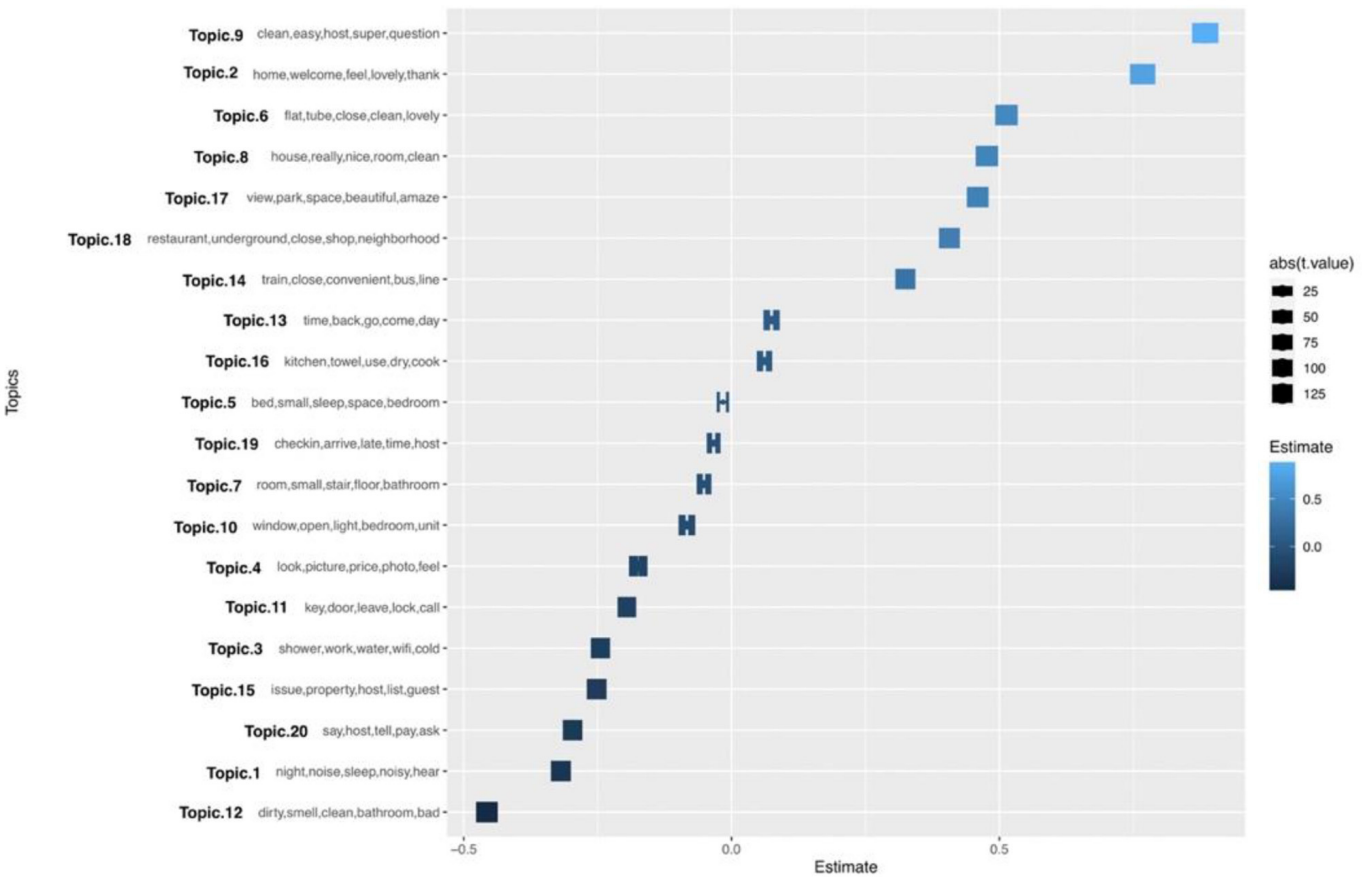
sLDA visualization. Source: created by the authors.

In Figure 10, the thickness of the topic bar represents the *t*-value, which indicates the statistical significance of the corresponding topic. The gradation of the topic color represents the estimated value, the lighter the color denotes a higher estimated score, and the darker color indicates a lower estimate score. Among topics with a positive coefficient, the higher the estimated coefficient of a topic on the x-axis, the higher the probability that satisfied Airbnb users talk about it. Among topics with negative coefficients, the lower the coefficient, the higher the probability that dissatisfied Airbnb users talk about it.

## Conclusion

### Discussion

In this study, LDA is employed to identify sources of Airbnb users' satisfaction and dissatisfaction. Different from previous Airbnb customer review studies that disregard the balance of positive and negative reviews in samples, this study used samples consisting of an equal number of positive and negative reviews. LDA was employed to extract latent topics from positive and negative reviews separately, contributing to differentiating satisfaction and dissatisfaction attributes. The results revealed 14 topics from positive reviews and 18 topics from negative reviews, supporting that the sources of dissatisfaction for customers are more diverse compared to the sources of satisfaction (Xu and Li, 2016). Except for “guest conflicts” and “room temperature,” the remaining topics identified in this study have also been reported in previous Airbnb studies. However, previous studies mainly reported the positive performance of certain attributes such as “communication” and “kitchen experience,” while overlooking the negative performance. Thus, this study provided a more comprehensive understanding of Airbnb service attributes.

As revealed by comparing LDA results from positive reviews and negative reviews, attributes under the dimension of tangibility are the major sources of Airbnb users' dissatisfaction. Those attributes associated with dysfunctional household equipment and poor room conditions are often discussed in negative reviews. However, the positive performance of those attributes in positive reviews was not often mentioned by Airbnb users, demonstrating that internal facilities related tangible attributes do not play a crucial role in formulating a satisfied lodging experience; this finding is consistent with Priporas et al. (2017). The attributes under the dimension of convenience are the major source of user satisfaction, consistent with Lee and Kim's (2018) finding that hedonic value is strongly associated with Airbnb users' satisfaction. However, in order to avoid Airbnb user dissatisfaction, it is important for hosts to provide an accurate description of the location of the property, because we found that complaints about unmatched descriptions are mainly related to the location. As for topics related to Airbnb hosts, both satisfied and dissatisfied Airbnb users emphasized interactions with Airbnb hosts, indicating that the behaviors of Airbnb hosts can be a driver of Airbnb user satisfaction and dissatisfaction. The relevant topics from positive reviews present the compliments to Airbnb hosts' positive behaviors, such as hosts' timely responses and assistance. Although a positive topic related to Airbnb hosts can also be observed in negative reviews, the remaining topics are all about complaints regarding Airbnb hosts, such as bad attitudes, late responses, and irresponsible behaviors.

In this study, sLDA is applied to determine the relative importance of attributes to Airbnb users' satisfaction and dissatisfaction. The statistical results of sLDA model reveal the different strength of service attributes to Airbnb users' satisfaction and dissatisfaction. More specifically, maintaining positive communication has the strongest predictive power to Airbnb user satisfaction. This could be related to that positive interpersonal communication with guest can help to foster initial trust in hosts (Gruber, 2020), which has a positive relationship with Airbnb user satisfaction (Liang et al., 2018). In contrast, poor room conditions such as dirty or smelly environment are the main driver of Airbnb user dissatisfaction. The topic “bed size and condition” is tested to be not significantly related to Airbnb users' satisfaction.

Furthermore, this study also extends previous studies by examining topic distributions in four types of Airbnb properties, contributing to revealing Airbnb users' different emphasis when they stay in different types of Airbnb properties. The findings show that Airbnb users who booked the entire property cared more about and convenience for exploring local communities, while those who stayed in the hotel room and shared room were more likely to encounter conflicts with other guests.

### Theoretical Implications

This study makes the following contributions to the literature. This is the first study to look separately at the attributes of customer satisfaction and dissatisfaction in the accommodation sector of the sharing economy. The findings support the hypotheses of Herzberg's two-factor theory in terms of the different nature of satisfaction and dissatisfaction attributes. Some findings of this study can provide a basis for the future development of questionnaires used for measuring Airbnb user satisfaction. For instance, two topics (“room temperature” and “guest conflicts”) that had not been reported in previous studies were identified. In particular, the emergence of “guest conflict” in this study can provide a valuable implication for future studies to examine interactions between different guests in a highly shared environment. This study also supports the existence of hybrid factors that can be both satisfiers and dissatisfiers (Kano et al., 1984). This study identified service attributes that can be categorized into a hybrid category, such as “communication” and “check in/out.”

This study extends the existing Airbnb studies that largely focus on the overall evaluation of Airbnb user experience (Cheng and Jin, 2019; Ju et al., 2019; Luo and Tang, 2019; Sutherland and Kiatkawsin, 2020) by comparing the emphasis level of service attributes discussed by users staying in four types of Airbnb properties. The results revealed Airbnb users' different expectations toward different types of Airbnb properties. The topic correlation analysis revealed the potential associations among different topics. In particular, the findings suggest the close connection between “home-like experience,” “help from hosts” and “revisit intention,” which can provide insights for future Airbnb loyalty studies.

From the perspective of methodology, LDA has been extensively applied in existing customer review studies while less attention has been paid to employing sLDA. This is the first study that employed sLDA to examine customer reviews in the lodging industry, and the results demonstrated the effectiveness of using sLDA to obtain insights from customer reviews into how different attributes affect customer satisfaction. Therefore, future studies can follow the same procedures of the present study by incorporating different outcome variables to identity topics with stronger predictive power to a specific variable.

### Practical Implications

This study has many valuable implications for Airbnb managerial practice. First, it revealed the heterogeneity of satisfaction and dissatisfaction attributes in Airbnb accommodation. The results can help Airbnb practitioners understand what aspects should be given more attention in order to increase guest satisfaction and avoid putting too much effort into the aspects that are not very valued by Airbnb users. For instance, Airbnb hosts need to emphasize on providing outstanding customer services in order to increase Airbnb user satisfaction. Besides, even though significant improvement in dissatisfiers (e.g., tangible features of the room) may not guarantee the increase of guest satisfaction, Airbnb hosts still have to maintain these aspects at an acceptable performance level to avoid guest dissatisfaction. As a successful business must not only create satisfaction, but also avoid dissatisfaction (Kim et al., 2016).

Second, the results from analyzing the topic distributions in four types of Airbnb properties suggest that Airbnb hosts renting shared and private rooms should develop measures in advance to avoid potential conflicts between different guests, as the topic “guest conflicts” appeared significantly more in these two types of Airbnb property. Third, the results of this study provide Airbnb practitioners valuable insights into priority setting when dealing with satisfaction and dissatisfaction attributes. For satisfiers, Airbnb hosts should give priority to improving communication experience of the guest, such as providing quick responses, and preparing useful pre-arrival instructions. On the other hand, hosts must ensure the cleanliness and hygiene of the room, especially the bathroom when taking measures to avoid dissatisfaction.

Forth, we also recommend Airbnb management to provide necessary training to Airbnb hosts regarding how to engage with Airbnb users in different situations and cater for their needs, considering that Airbnb hosts are regarded as the key contributor to Airbnb users' evaluation. Different from traditional hotel staff, they are generally trained to provide standardized service to guests. Some Airbnb hosts may not have been engaged in the service industry, resulting in their lack of service awareness and making them unable to facilitate a satisfactory lodging experience to Airbnb users. Last, the identified attributes that reveal customers' expectations can be used as useful references for Airbnb and hosts to create social media content that is of value to customers, which can contribute to the building of trust and confidence in the platform (Martínez-Navalón et al., 2019).

### Limitations

Although using the lexicon-based approach to conduct sentiment analysis can achieve sufficiently accurate results for academic research (Pang and Lee, 2008), the sentiment lexicon used in this study is not exclusively designed for the lodging industry, resulting in inaccurate sentiment scoring to certain opinion words. Hence, it is encouraged for future studies to use the sentiment lexicon with context emphasizing the lodging industry to achieve better results. Besides, this study only examined how Airbnb users' perceptions to different service attributes vary in different types of Airbnb property, making it insufficient to explain Airbnb users' behaviors. Future studies can examine Airbnb users' perceptions by looking at some other perspectives, such as Airbnb users' cultural backgrounds, the purpose of stay, and price ranges of the property. Furthermore, the inclusion of data from 12 different countries for this study can improve the generalizability of the results. However, some drivers of Airbnb users' satisfaction or dissatisfaction in certain countries could be overlooked. Consequently, future studies could examine Airbnb reviews in a region or country that has not been explored to identify some context-specific service attributes.

## Data Availability Statement

Publicly available datasets were analyzed in this study. This data can be found at: http://insideairbnb.com/get-the-data.html.

## Author Contributions

KD: conceptualization, methodology, original draft preparation, software, and revision writing. WC: validation, reviewing, and editing. KN: software, data curation, and text mining. SN: investigation, reviewing, and editing. PS: revision writing, visualization, and editing. All authors have read and agreed to the submitted version of the manuscript.

## Conflict of Interest

The authors declare that the research was conducted in the absence of any commercial or financial relationships that could be construed as a potential conflict of interest.
