# Supplementary material for: Exploring Sources of Satisfaction and Dissatisfaction in Airbnb Accommodation Using Unsupervised and Supervised Topic Modeling
Source: Front Psychol. 2021 Apr 21;12:659481. doi: 10.3389/fpsyg.2021.659481 (PMC8096999; doi:10.3389/fpsyg.2021.659481)
Supplement: Supplementary file 1 [file Table_1.DOCX]

**Appendix B.** Top topic words

| **Positive reviews** | | | | | | | | | | | | | | |
| --- | --- | --- | --- | --- | --- | --- | --- | --- | --- | --- | --- | --- | --- | --- |
| **Topic.1** | | **Topic.2** | | **Topic.3** | | **Topic.4** | | | **Topic.5** | | **Topic.6** | | **Topic.7** | |
| night | | give | | flat | | kitchen | | | location | | Day | | beautiful | |
| floor | | host | | bus | | bed | | | perfect | | arrive | | view | |
| bite | | help | | central | | use | | | host | | leave | | space | |
| window | | experience | | area | | shower | | | look | | late | | amaze | |
| door | | time | | lovely | | bathroom | | | need | | time | | love | |
| issue | | helpful | | tube | | bedroom | | | definitely | | book | | park | |
| open | | local | | quiet | | cook | | | highly | | early | | enjoy | |
| problem | | see | | comfortable | | wifi | | | excellent | | take | | wonderful | |
| sleep | | offer | | easy | | towel | | | extremely | | meet | | decorate | |
| noise | | kind | | close | | work | | | comfortable | | arrival | | relax | |
| **Topic.8** | | **Topic.9** | | **Topic.10** | | **Topic.11** | | | **Topic.12** | | **Topic.13** | | **Topic.14** | |
| neighborhood | | room | | make | | restaurant | | | nice | | easy | | house | |
| locate | | small | | home | | underground | | | really | | location | | time | |
| area | | people | | feel | | shop | | | clean | | check_in | | back | |
| city | | space | | welcome | | close | | | super | | clean | | come | |
| quiet | | big | | comfortable | | location | | | need | | question | | family | |
| safe | | bathroom | | lovely | | many | | | friendly | | need | | day | |
| street | | enough | | breakfast | | distance | | | enjoy | | quick | | love | |
| restaurant | | clean | | sure | | nearby | | | comfortable | | communication | | next | |
| build | | price | | warm | | cafe | | | host | | host | | thank | |
| right | | find | | experience | | away | | | helpful | | respond | | visit | |
| **Negative reviews** | | | | | | | | | | | | | | |
| **Topic.1** | **Topic.2** | | **Topic.3** | | **Topic.4** | | **Topic.5** | **Topic.6** | | **Topic.7** | | **Topic.8** | | **Topic.9** |
| flat | look | | window | | kitchen | | night | really | | close | | host | | say |
| issue | picture | | unit | | use | | noise | nice | | location | | book | | bad |
| property | list | | cold | | towel | | sleep | location | | restaurant | | pay | | leave |
| however | photo | | open | | provide | | street | bite | | area | | day | | tell |
| guest | disappoint | | hot | | dry | | noisy | quite | | locate | | hotel | | experience |
| problem | show | | heat | | clean | | morning | overall | | underground | | refund | | ask |
| owner | review | | park | | wash | | hear | okay | | away | | money | | want |
| experience | find | | night | | cloth | | loud | price | | neighborhood | | offer | | never |
| unfortunately | description | | fan | | thing | | next | little | | train | | another | | know |
| arrival | different | | air | | cook | | every | however | | shop | | night | | thing |
| **Topic.10** | **Topic.11** | | **Topic.12** | | **Topic.13** | | **Topic.14** | **Topic.15** | | **Topic.16** | | **Topic.17** | | **Topic.18** |
| feel | door | | shower | | bedroom | | room | clean | | host | | bed | | arrive |
| home | build | | water | | break | | house | dirty | | wifi | | small | | time |
| time | leave | | day | | light | | guest | smell | | message | | floor | | check_in |
| make | open | | problem | | wall | | people | bed | | call | | space | | late |
| family | feel | | work | | bathroom | | bathroom | sheet | | send | | people | | day |
| long | key | | bathroom | | old | | use | bathroom | | never | | sleep | | hour |
| want | lock | | toilet | | kitchen | | bad | bad | | contact | | stair | | leave |
| travel | come | | fix | | live_room | | share | hair | | find | | uncomfortable | | wait |
| need | someone | | use | | need | | clean | stain | | phone | | mattress | | tell |
| spend | enter | | take | | floor | | really | floor | | respond | | sofa | | key |

**Appendix C.** SLDA topic summary.

| **Topic No.** | **Topic Name** | **Top Words** |
| --- | --- | --- |
| Topic.1 | Noise | night, noise, sleep, street, noisy, morning, hear, loud, people, next |
| Topic.2 | Home-like experiences | home, feel, make, welcome, lovely, thank, wonderful, host, comfortable, breakfast |
| Topic.3 | Shower issues | shower, work, water, wifi, day, issue, cold, problem, fix, hot_water |
| Topic.4 | Unmatched description | look, picture, price, photo, location, feel, expect, hotel, find, bite |
| Topic.5 | Bed size and condition | bed, small, sleep, space, people, bedroom, family, sofa, uncomfortable, kid |
| Topic.6 | Easy access to desired places | flat, location, clean, close, tube, need , quiet, nice, central, lovely |
| Topic.7 | Room size | room, small, floor, bathroom, stair, people, luggage, quite, bite, toilet |
| Topic.8 | Room condition | house, really, nice, clean, room, host, time, kind, feel, big |
| Topic.9 | Communication | clean, host, location, easy, need, helpful, super, question, definitely, comfortable |
| Topic.10 | Room temperature | window, light, bedroom, open, unit, hot, door, fan, close, air |
| Topic.11 | Door lock and key | door, key, leave, go, lock, find, give, come, call, say |
| Topic.12 | Dirtiness and smells | dirty, clean, smell, bathroom, bad, floor, bed, sheet, wall, stain |
| Topic.13 | Revisit intention | time, go, back, come, day, make, want, trip, spend, take |
| Topic.14 | Public transportation | close, train, location, convenient, area, away, line, bus, restaurant, subway |
| Topic.15 | Property issues | issue, host, property, guest, list, however, make, experience, problem, owner |
| Topic.16 | Kitchen experience | kitchen, use, towel, dry, provide, cook, cook, clean, need, fridge, cloth |
| Topic.17 | View | view, park, space, beautiful, amaze, area, location, perfect, city, love |
| Topic.18 | Neighborhood environment | restaurant, underground, close, location, neighborhood, shop, bar, perfect, street, locate |
| Topic.19 | Check in and out | check_in, time, host, arrive, late, day, hour, checkout, leave, message |
| Topic.20 | Host’ irresponsible behavior | host, say, book, tell, ask, leave, pay, bad, day, experience |
