# Supplementary figures and images for: Exploring Sources of Satisfaction and Dissatisfaction in Airbnb Accommodation Using Unsupervised and Supervised Topic Modeling
Source: Front Psychol. 2021 Apr 21;12:659481. doi: 10.3389/fpsyg.2021.659481 (PMC8096999; doi:10.3389/fpsyg.2021.659481)

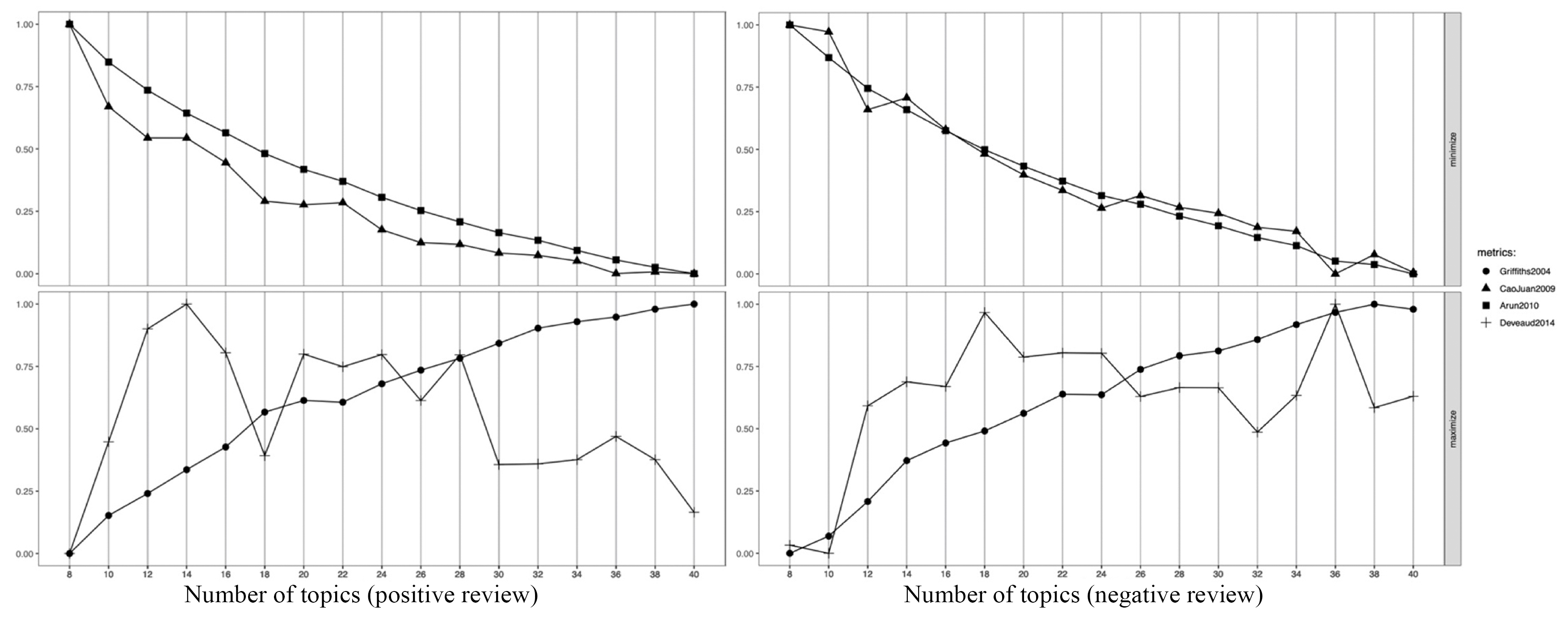

Supplement: Supplementary file 2 [file Image_1.JPEG]
